# Optimization of a Calcium-Based Treatment Method for Jellyfish to Design Food for the Future

**DOI:** 10.3390/foods11172697

**Published:** 2022-09-04

**Authors:** Francesca Anna Ramires, Stefania De Domenico, Danilo Migoni, Francesco Paolo Fanizzi, Dror L. Angel, Rasa Slizyte, Katja Klun, Gianluca Bleve, Antonella Leone

**Affiliations:** 1 Unit of Lecce, National Research Council–Institute of Sciences of Food Production, 73100 Lecce, Italy; 2 Department of Biological and Environmental Sciences and Technologies (DiSTeBA), University of Salento, 73100 Lecce, Italy; 3 Department of Maritime Civilizations, School of Archaeology and Maritime Cultures, University of Haifa, Haifa 31905, Israel; 4 Department of Fisheries and New Biomarine Industry, SINTEF Ocean, Brattørkaia 17C, 7010 Trondheim, Norway; 5 National Institute of Biology, Marine Biology Station, 6330 Piran, Slovenia; 6 Local Unit of Lecce, Consorzio Nazionale Interuniversitario per le Scienze del Mare (CoNISMa), 73100 Lecce, Italy

**Keywords:** jellyfish, novel food, safety, quality, nutritional traits, organic calcium salts

## Abstract

Edible jellyfish are a traditional Southeast Asian food, usually prepared as a rehydrated product using a salt and alum mixture, whereas they are uncommon in Western Countries and considered as a novel food in Europe. Here, a recently developed, new approach for jellyfish processing and stabilization with calcium salt brining was upgraded by modifying the pre-treatment step of freshly caught jellyfish and successfully applied to several edible species. Treated jellyfish obtained by the application of the optimized version of this method respected both quality and safety parameters set by EU law, including no pathogenic microorganisms, absence or negligible levels of histamine and of total volatile basic nitrogen, no heavy metals; and the total bacterial, yeast, and mold counts were either negligible or undetectable. Jellyfish treated by the presented method exhibited unique protein content, amino acid and fatty acid profiles, antioxidant activity, and texture. The optimized method, initially set up on *Rhiszostoma pulmo*, was also successfully applied to other edible jellyfish species (such as *Cotylorhiza tuberculata*, *Phyllorhiza punctata*, and *Rhopilema nomadica*) present in the Mediterranean Sea. This study discloses an innovative process for the preparation of jellyfish-based food products for potential future distribution in Europe.

## 1. Introduction

Jellyfish (JF) are mainly available and consumed as food in Asian countries. However, their use in food preparation has recently spread widely worldwide in the form of ready-to-use products, also attributed to the availability on internet market channels [1,2]. In recent years, JF food products have also become more popular in Western Countries [3], possibly due to an increase in JF populations related to environmental factors, such as rising temperatures, marine pollution, oxygen depletion, and a reduction of marine predator populations [4].

Jellyfish blooms and the invasive behavior of some species make them a good candidate for potential resources for food and other applications [5,6,7].

JF for human consumption are generally prepared by separating the umbrella from the oral arms and washing them extensively in order to eliminate mucus, gonads, sand, and superficial microorganisms. Then, these highly perishable JF tissues are treated with mixtures of NaCl and aluminum salts (alum) [1,8] in order to stabilize them, thus extending their shelf life, reducing any microbial issues, and promoting the expected organoleptic characteristics and texture so highly appreciated by Eastern people. In Asia, although ancient recipes and empirical procedures are still followed [9], new methods still based on the use of alum have been developed [1], since Eastern cuisines pay high attention to product texture and taste. However, the traditionally preserved JF products available on the market contain high levels of aluminum, which is strongly bound to the tissue [10] and cannot be eliminated through the usual washes applied before consumption.

The research on new JF stabilization procedures and treatments for food uses is recently moving to limit the use of alum, due to its toxicity [11,12], and to obtain semi-finished and finished products closer to Western Countries’ style and expectations. Pedersen et al. [13] reported the possible substitution of alum with other tanning salts, such as iron salts, with a mechanism similar to a tanning process. In addition, the same authors produced alum-free crisps by soaking the jellyfish in ethanol and drying it afterward.

At present, JF is a novel food in Europe [14] and is limited by several issues, such as the (*i*) very high aluminum content of Asian traditionally preserved JF products and *(ii*) lack of safe stabilization methods for treating and processing JF tissues according to EU safety standards. Consequently, the development of a new, safe, and validated technology for processing JF could encourage regulatory authorities to approve the use and commercialization of edible JF species.

In our previous work, we proposed new parameters for the risk assessment of JF as food in Europe [15]. They were newly identified and applied to JF and JF-derived products not already included in the European regulation on seafood safety [16,17]. More recently, we proposed a new procedure [18] to process JF raw materials using calcium salts, which were selected from the food additives allowed in several Western Countries (the EU, Australia, USA, and New Zealand). It was observed that calcium salts were able to work as firming and stabilizing agents for JF biomasses, thus opening the opportunity to prepare safe semi-finished products suitable for subsequent food applications. Bleve et al. [18] set up the procedure under controlled conditions, thus demonstrating the microbiological safety of the method.

In this paper, the above-mentioned method was further optimized by modifying the JF pre-treatment step and including washes with sterile seawater; furthermore, the whole procedure was validated by using several JF species with different characteristics. Two different strategies are described here in order to substitute the use of sterile seawater for JF washing, with this step being quite challenging and not applicable on an industrial scale. Additionally, JF treated with calcium salts were tested for safety and quality aspects—the treated tissues were analyzed for protein, fatty acids, amino acids, element content, and antioxidant activity. The process efficacy was initially tested on the model species *Rhizostoma pulmo* and successively verified on three other potentially edible JF species (*Cothyloriza tuberculata*, *Phylloriza punctata*, and *Rophilema nomadica*). A comparison of the JF treated with the optimized processing method proposed here with JF prepared with the traditional Asian methods was also carried out.

## 2. Materials and Methods

### 2.1. Sample Collection and Pre-Treatment

*Rhizostoma pulmo*, Macrì 1778 (Cnidaria, Scyphozoa, Rhizostomatidiae) specimens were hand-collected from a motorboat in the Ionian Sea (Ginosa Marina, Italy 40°24′37.5″ N 16°53′04.2″ E) using a nylon landing net (3.5 cm mesh size) during samplings in the 2019–2020 summer period and processed by either of the two procedures described below.

In the absence of specific slaughter guidelines for cnidarians [19], the traditional method used in Asian Countries to kill the jellyfish [1] was applied to *R. pulmo* specimens by cutting the oral arms from the umbrella and removing the gastric content. For *Cotylorhiza tuberculata*, *Phyllorhiza punctata*, and *Rhopilema nomadica* samples, also sampled during the 2019–2020 summer period, whole JF were immediately frozen at −40 °C. At least 5 different specimens from each species were used; for *Rhizostoma pulmo*, randomized sampling was conducted as in Leone et al. [20].

**Figure 1:**
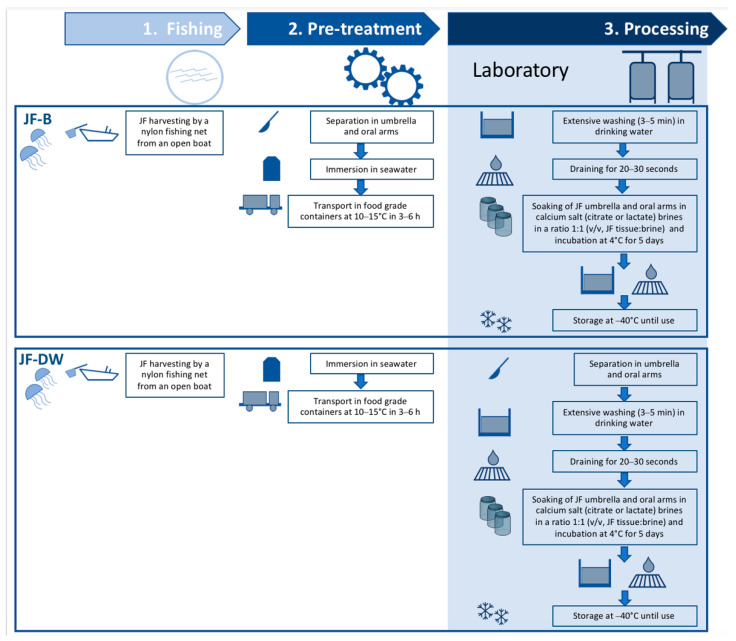
Diagram illustrating the procedure of the JF treatment procedures.

All the procedures followed in this study are summarized in the scheme reported in Figure 1.

*Procedure 1 (JF-B)*. JF oral arms were separated from the umbrellas, immersed in refrigerated seawater, and transported to the laboratory (max 3–6 h). At the laboratory, jellyfish parts were extensively washed for 3–5 min in drinking water.

*Procedure 2 (JF-DW)*. Whole JF were immersed in refrigerated seawater immediately after capture and transported to the laboratory (max 3–6 h). At the laboratory, the jellyfish were immersed in drinking water, the umbrellas were separated from the oral arms, and these parts were extensively washed for 3–5 min.

Following both procedures, the umbrellas and oral arms were placed in sterile food-grade plastic bags and stored at −80 °C or immediately treated in the newly formulated brines with a firming agent.

Procedure 2 (JF-DW) was also applied to *Cotylorhiza tuberculata* samples harvested from the Gulf of Trieste (northern Adriatic Sea, Slovenia) and the Ionian Sea (Italy), and to *Rhopilema nomadica* and *Phyllorhiza punctata* harvested from the Eastern Mediterranean (Israeli coastal waters), frozen at −40 °C, and shipped on dry ice to Italy. Frozen material was thawed overnight on ice and then extensively washed with drinking water.

Aliquots of all untreated and treated JF samples (see below) were freeze-dried in order to analyze the elemental, lipid, and amino acid compositions or immediately frozen to evaluate the protein content and antioxidant activity. A commercial product, jellyfish stored in brine, from Japan (Salt-Alum Jp) was also analyzed and used for comparison.

### 2.2. Microbiological Analyses of Pre-Treated Jellyfish

Ten grams from each JF sample were added to 90 mL of buffered peptone water (Biolife Italiana, Milano, Italy) as a diluent (1:10). For total bacterial counts (TBCs), samples were diluted and plated by the pour plate technique on plate count agar (PCA) (Biolife Italiana, Milano, Italy) at pH 7.0 and incubated at 30 °C for 72 h; the enumeration of yeast and molds was performed by incubation at 25 °C for 5 days on dichloran Rose–Bengal chloramphenicol agar (DRBC, Thermo Fisher Scientific, Monza, Italy). The presence of Enterobacteriaceae, *Escherichia coli*, *Salmonella enteritidis*, Coagulase-positive staphylococci, *Staphylococcus aureus*, *Vibrio* spp. (*V. cholerae*, *fluvialis*, *parahemolyticus*, and *vulnificus*), *Bacillus* spp. (*B. cereus*, *turigensis*, *megaterium*, and *subtilis*), *Shewanella putrefacens*, *Aereomonas hydrophila*, and *Pseudomonas fluorescens* was assessed following the procedure described by Bleve et al. [18].

For the determination of halophilic microorganisms, JF samples were homogenized with a sterilized blender, and 25 g of each sample was added to peptone seawater 0.1% (*w*/*v* peptone) and artificial seawater. All samples and their respective serial dilutions were plated in different media dissolved in artificial seawater as described by Bleve et al. [15,18]. For each plate, the number of colony-forming units (CFU) per gram of JF was determined.

The JF samples were also submitted to an accredited external laboratory for independent analyses (Laboratori Artas Società Cooperativa, Poggiardo, Lecce, Italy). Ten grams of each JF sample were added to 90 mL of buffered peptone water (Biolife Italiana, Milano, Italy) as a diluent (1:10) and homogenized for 2 min in a Stomacher in accordance with specific standard methods for the total bacterial count (UNI EN ISO 4833-1:2013), coliforms (ISO 4832:2006), β-glucuronidase-positive *Escherichia coli* (ISO 16649-2:2001), coagulase-positive staphylococci (UNI EN ISO 6888-2:1999), and yeast and molds (ISO 21527-1:2008, ISO 21527-2:2008). For the detection of the pathogenic bacteria *Salmonella* spp. (UNI EN ISO 6579-1:2017) and *Listeria monocytogenes* (ISO 11290-1:2017), 25 g of jellyfish samples were suspended in 225 mL of buffered peptone water (Biolife Italiana, Milano, Italy) and Fraser broth at half concentration (Biomerieux, Marcy l’Etoile, France), respectively, as diluents.

### 2.3. Jellyfish Treatment in Brine

Solutions of calcium citrate and calcium lactate were prepared using 0.1 M calcium-citrate solution or 0.1 M calcium–lactate hydrate solution and adjusted to pH 5.0 by using the corresponding alpha organic acids, 1 M citric acid or 1 M lactic acid 85% (*v*/*v*), all from Sigma-Aldrich (Darmstadt, Germany). These concentrations were arbitrarily chosen and tested for JF stabilization treatment, as already previously described by Bleve et al. [18].

In order to determine these treatment conditions, JF tissue samples were soaked in calcium salt brines at different pH values ranging from 3 to 6 for 10 days. A starting brine pH value of 5 was determined as the best compromise, obtained after the evaluation of the effects produced by calcium salts on JF tissue’s traits, such as texture and appearance, and safety aspects. More acidic pH values (<5) exerted undesirable effects on JF consistency by damaging (attacking and corroding) the tissue (data not shown).

Pre-treated jellyfish (umbrella and oral arms washed for 3–5 min in drinking water, JF-B, or JF-DW) were immersed in brines at a 1:1 ratio (*v*/*v*, JF tissue:brine) in food-grade glass or plastic containers. These technological phases were arbitrarily transferred from the conditions generally used for vegetable stabilization treatment and directly tested for the first time for their possible adaptation to JF tissues. The containers were stored at 4 °C for 5 days and then JF tissues (umbrella and oral arms) were removed, washed with drinking water to eliminate excess salts, sealed in food-grade plastic bags, and stored at −80 °C for further tests. Aliquots of each sample were also freeze-dried and stored.

### 2.4. JF Treatment with NaCl and Aluminum Salt

*R. pulmo* specimens were also processed by the traditional Asian method [21,22] using salt and alum (Salt–Alum JF-DW). Briefly, *R. pulmo* umbrellas were separated from the oral arms and extensively washed for 3–5 min with drinking water. The washed umbrellas were covered with a salt mix containing 90% (*w*/*w*) NaCl and 10% KAl(SO_4_)_2_·12H_2_O (alum) (*w*/*w*) (Cruciani Prodotti Crual, Roma, Italy) using about 100 g of salt–alum mix per 1 kg of JF biomass and incubated at 4 °C in a food-grade glass container. After 4 days, brines released from the JF tissues were removed and the umbrellas were covered with a salt mixture containing 92.5% (*w*/*w*) NaCl and 7.5% alum (*w*/*w*). After 4 days, the same procedure was repeated, but the percentage of alum in the salt mix was reduced to 5% (*w*/*w*) and finally to 2.5% (*w*/*w*). At the end of the process, the salted jellyfish samples were left to dry on a draining rack at room temperature for 4 days, inverting them several times to drain and remove excess water. The entire process took 20 days. Aliquots of each sample were also lyophilized.

### 2.5. Physical–Chemical Analyses

The histamine concentration in the JF was determined according to the AOAC N° 021402 2014 method (HistaSure ELISA, LDN, Germany). The total volatile base nitrogen (TVBN) was determined by treating each jellyfish sample (100 g) with 0.6 M perchloric acid (Merck KGaA, Darmstadt, Germany). After alkalinization, the extract was exposed to steam distillation and an acid receiver absorbed the volatile base components. The TVBN concentration was determined by the titration of the absorbed bases [23].

For salinity and pH determination, 15 g of JF tissue was collected and stored at −80 °C for further analysis. The texture was measured as described in Bleve et al. [18], using a digital penetrometer (model 53205, TR Turoni, Srl Forlì, Italy). The penetration test was performed using a three-bar probe (3 × 22 mm) for a total plunger area of 1.98 cm^2^ by operating on samples consisting of radial triangular slices of the JF tissues. Firmness values were reported as the means of three different measures, expressed in Newtons (N). All analyses were carried out in triplicate.

### 2.6. Elemental Analyses

The elemental composition (Al, As, B, Ba, Ca, Cd, Cr, Cu, Fe, Hg, K, Mg, Mn, Na, Ni, Pb, Sr, V, and Zn) of the JF samples was measured using inductively coupled plasma–atomic emission spectroscopy (ICP-AES). Lyophilized JF samples were weighed and mixed with 4 mL of H_2_O_2_ and 6 mL of super-pure HNO_3_ 69%, digested at 180 °C for 10 min using a microwave digestion system (START D, Milestone Srl, Sorisole (BG), Italy), cooled, diluted with super pure water, and filtered through 0.45 μm syringe filters. A spectrometer (Thermo Fisher Scientific, iCap 6000 Series, Monza, Italy) was previously calibrated for quantitative analysis with five standard solutions containing known concentrations of the elements (0.001, 0.01, 0.1, 0.5, and 1.0 mg/L). The calibration lines showed correlation coefficients (r) greater than 0.99 for all the measured elements. The analysis results were expressed as the average (+/− standard deviation of three different measurements) element concentrations, expressed in ppm (mg/kg of sample weight). JF supernatants obtained after the centrifugation of the JF samples were also analyzed, corresponding to the lyophilized samples.

### 2.7. Protein Content Determination

Jellyfish samples were homogenized in a blender and diluted in distilled water until a homogeneous solution was obtained. The total protein content in each sample was evaluated using the Bradford assay [24], set for an Infinite 200 PRO microplate reader (TECAN, Männedorf, Switzerland) and using bovine serum albumin (BSA) as a standard.

### 2.8. Antioxidant Activity Determination

In the diluted JF samples (protein assays), the antioxidant activity (AA) was evaluated using the Trolox Equivalent Antioxidant Capacity (TEAC) method based on the radical cation ABTS•+ and Trolox as a standard. The assay was adapted for an Infinite 200 PRO microplate reader (TECAN, Männedorf, Switzerland), and both samples and the standard were assayed as described in De Domenico et al. [25]. The results were expressed in nmol of Trolox equivalents per gram of JF fresh weight (nmol TE/g FW).

### 2.9. Lipid Extraction

Total lipids were extracted using a modified Bligh and Dyer method [26]: lyophilized samples (200 mg) were mixed with 12 mL of a solution of chloroform:methanol (2:1) and 3 mL KCl (0.88%), shaken, and centrifuged at 5140× *g* for 5 min. The lower phase was set aside, and the upper phase was subjected to further extraction with one volume of a solution of chloroform:methanol (2:1, *v*/*v*). After phase separation, the lower phase was isolated and added to the first one, and mixed with one-quarter volume of a solution of methanol:water (1:1, *v*/*v*). The lower phase was dried using nitrogen and analyzed for lipid composition.

#### Fatty Acids Analysis

Fatty acid methyl esters (FAME) were obtained according to Leone et al. [27] using boron trifluoride (BF_3_), as follows. The total lipid extract in hexane (200 μL) was saponified at 90 °C for 20 min with 0.5 M KOH in methanol (3 mL) with a known quantity of internal standard (methyl-tricosanoate). Fatty acids were methylated with 2 mL of BF_3_ in methanol (14%), and the samples were evaporated under a stream of nitrogen and dissolved in 50 μL of hexane, and 1 μL was analyzed by gas chromatography–mass spectrometry (GC-MS). GC–MS analyses were performed using an AGILENT 5977E gas chromatograph (Agilent Technologies, Santa Clara, CA, USA) on a VF-WAXms (60 m, 0.25 mm i.d., 0.25 mm film thickness, Agilent) with the following parameters: the column temperature was maintained at 160 °C for 1 min, programmed at 4 °C/min to 240 °C for 30 min. Helium was used as a carrier gas at a constant flow rate of 1 mL/min. The mass spectrometer was operated in the electron impact mode with a scan range of 50–700 m/z. The temperature of the MS source and quadrupole were set at 230 °C and 150 °C, respectively. Analyses were performed in the full-scan mode. Compounds were identified by comparing the retention times of the chromatographic peaks with those of authentic standards (F.A.M.E. Mix C8-C24, Sigma-Aldrich Corporation, St. Louis, MO, USA) analyzed under the same conditions. The MS fragmentation patterns were compared with those of pure compounds, and a mass spectrum database search was performed using the National Institute of Standards and Technology (NIST) MS 98 spectral database.

### 2.10. Amino Acid Analysis

The amino acid profile in lyophilized samples was analyzed with an HPLC system (Agilent Infinity 1260, Agilent Technologies) coupled with an online post-column derivatization module (Pinnacle PCX, Pickering Laboratories, Mountain View, CA, USA), using ninhydrin (Trione) as a derivatizing reagent and a Na^+^ ion-exchange column (4.6 × 110 mm, 5 μm). Eighteen standard amino acids, ammonia, and taurine were quantified from standard curves measured with the amino acid standards. Prior to the analysis, the samples were hydrolyzed in 6 M HCl containing 0.4% mercaptoethanol for 24 h at 110 °C (HCl hydrolysis). Glutamine and asparagine were converted to glutamic and aspartic acid, respectively. Cysteine (Cys) was quantified as cystin (Cys-Cys). The samples were filtered via a micro filter, the pH was adjusted to 2.2, and the samples were further diluted with a citrate buffer (pH 2.2) for HPLC analysis.

### 2.11. Statistical Analysis

All data presented are the means of three independent replicates (*n* = 3). Statistical analysis was based on one-way analysis of variance. Tukey’s post hoc method was applied to establish significant differences among the means (*p* < 0.05, *p* < 0.01 and *p* < 0.001). All statistical comparisons were performed using Sigma-Stat, version 3.11 (Systat Software Inc., Chicago, IL, USA).

Statistical analyses on the protein content and antioxidant activity were performed in Graphpad Prism 6.0 using an analysis of variance (ANOVA) followed by Dunnett’s multiple comparison post hoc test to compare each treatment with the control, and Bonferroni’s multiple comparison post hoc test to compare the samples with each other. Differences were considered statistically significant for *p* values of < 0.05. All assays were replicated (*n* = 3) and data were represented as the mean ± standard deviation (SD). Principal component analysis (PCA) to compare important physical parameters and chemical compounds associated with the samples was carried out using XLSTAT software (Addinsoft Inc., Long Island City, NY, USA).

## 3. Results and Discussion

### 3.1. Safety Traits of Rhizostoma Pulmo JF Treated Samples

Two pre-treatment strategies are described here in order to set up an optimized stabilization method for jellyfish intended for food use. In particular, in the first approach (JF-B, procedure 1), jellyfish were immediately washed with refrigerated seawater just after being harvested, and their umbrellas were promptly separated from their oral arms, an operation that can be carried out on the freshly caught jellyfish directly on board. This approach can greatly reduce the ashore disposal of possibly large quantities of JF by-products, thus returning unused JF material directly to the sea. In the second approach (JF-DW, procedure 2), whole JF were transported in chilled seawater to the laboratory, where the use of drinking water was tested for washing JF after transport.

This study proposes the combination of washing with drinking water, a procedure commonly used in the fishing industry, and subsequent treatment with calcium salts. It was observed that this method helped to stabilize the JF tissues, to improve their texture and nutraceutical traits, and to reduce undesired microorganisms in the processed JF products. Moreover, this approach represents an optimization of the recently proposed method for stabilizing and processing JF as food products for human consumption [18].

The main phases of the two procedures for JF preparation proposed in this study are presented in Figure 1 from the starting material to the final products.

In both pre-treatments proposed in this article, JF samples were washed with drinking water, although at different times. In the JF-B procedure, this washing step occurred after cleaning, cutting, and storing the animals in seawater, and before placing them in calcium salt solutions in the laboratory; in the JF-DW approach, instead, whole JF were firstly transported to the laboratory in chilled seawater and were then immersed in drinking water for the time necessary to wash and prepare the JF tissues before soaking them in calcium salt solutions.

At the starting point, both JF-B and JF-DW pre-treatments ensured negligible levels of JF-associated microorganisms, also in terms of halophilic microbes (Tables S1 and S2). This evidence demonstrates a better ability of both JF-B and JF-DW pre-treatments in reducing the initial JF microbial load compared with the seawater treatment (JF-SW) used in Bleve et al. [18]. Moreover, the use of calcium lactate and calcium citrate brines prevented the growth of potential pathogens (*Vibrio* spp., *Salmonella* spp., *Listeria monocytogenes*, and staphylococci) and spoiling microbial contaminants in both JF-B and JF-DW (Tables S1 and S2). The latter evidence was verified by applying the accredited standard parameters established by the law in force for food safety and process hygiene criteria to the Ca-Lactate and Ca-Citrate *R. pulmo* samples treated with both JF-B and JF-DW methods [15]. The approach with calcium salt brines had already been explored by Bleve et al. [18], where calcium lactate E327 and calcium citrate E333 were successfully tested on the edible JF species *R. pulmo*, being both included among the list of food additives and firming agents permitted in the European Union, U.S.A., Australia, and New Zealand.

The total counts of staphylococci were acceptable and showed a similar trend in all JF samples. *Escherichia coli*, coliforms, yeast, molds, and the pathogens *Salmonella* spp. and *L. monocytogenes* were not detected in any of the tested samples treated with either the JF-B or JF-DW methods (Table 1).

Several other studies have reported the presence of both bacteria [28] and fungi associated with JF tissues (body and mucus), which may present a risk to humans [15].

Histamine and TVNB were not detected in the tested JF samples (<3 mg/Kg and <0.1 mg/100 g, respectively) (Table 1), thus indicating that there was no tissue degradation and also confirming that the used procedure maintained the freshness of the JF raw material.

In order to compare this optimized process with the traditional Asian procedure, a batch of *R. pulmo* was treated in parallel using mixtures of NaCl and alum for tissue stabilization (Salt-Alum JF-DW) as described by Hsieh et al. [8] and Pedersen et al. [13]. The Salt-Alum JF-DW samples exhibited low counts of *Bacillus* spp. (4 × 10^1^ CFU/g) and discrete levels of halophilic bacteria (4.4–9.9 × 10^2^ CFU/g) and yeasts (2 × 10^2^–10^3^ CFU/g), although no microbial pathogens were detected (Table S3).

Additional tested parameters [15,18], including the total bacterial count, yeasts, Enterobacteriaceae, *Vibrio* spp., coagulase-positive staphylococci, and *Bacillus* spp., indicated that the Salt-Alum JF-DW samples were safe for consumption.

### 3.2. Chemical–Physical Characteristics of Treated R. pulmo Samples

The two proposed pre-treatments (JF-B and JF-DW) exerted different effects on the texture, pH, and salinity of the obtained samples (Table 2).

The optimized calcium citrate and calcium lactate brine treatments exerted different effects on the chemical–physical features of the JF tissues. The preliminary results reported here showed that salt treatment in the JF-B samples reduced the tissue texture (in terms of penetration force); moreover, salinity values of around 2% were measured and the pH values were very different between the two calcium treatments (Table 2). In Ca-Citrate and Ca-Lactate JF-DW samples instead, both brine preparations increased the tissue texture, achieving values of 1.8 and 1.6-fold, respectively, higher than the untreated samples. Additionally, these samples showed reduced salinity values and pH values equal to the initial ones (Table 2). As a result, the JF-DW procedure was selected as the preferred pre-treatment method for further experiments.

Lee et al. [29] already demonstrated the ability of calcium-based food additives (including calcium acetate, calcium carbonate, calcium–casein, calcium chloride, calcium citrate, calcium lactate, calcium sulfate, and calcium phosphate) to improve gelation and polymerization, as occurs during the preparation of surimi from codfish. The increased texture in both Ca-Citrate and Ca-Lactate JF-DW samples, in terms of higher penetration force, can be considered a good index of quality, since those products became denser and more manageable for the subsequent steps. However, Ca-Citrate and Ca-Lactate JF-DW samples showed a gel-like consistency very different from the rubbery and elastic texture of Salt-Alum JF-DW produced following the traditional Asian method. The pH was maintained at around 5 in the Ca-Citrate-treated JF-DW, whereas higher pH levels were obtained in the Ca-Lactate samples. Although being higher than those of the Salt-Alum JF-DW samples, these pH values ensured the expected safety level requested for the semi-finished product (as already shown in Table 1 and Table S1). The appearance of both semi-finished products obtained either by the methods proposed here (Ca-Citrate and Ca-Lactate JF-DW) and by Salt-Alum JF-DW are shown in Figure S1. The two JF-B and JF-DW pre-treatments exerted opposite effects on the texture of the Ca-Citrate- and Ca-Lactate-treated samples in comparison with the corresponding JF-SW samples [18]. In fact, the texture (in terms of penetration force) increased in the JF-DW samples, whilst it decreased in the JF-B samples. The latter evidence seems to indicate that prolonged exposure of JF tissues to drinking water during the JF-B procedure could affect their structure. Regarding the pH values, the JF-B samples showed values very close to those of JF-SW samples, thus evidencing a significant difference with respect to the samples treated with either of the two calcium salts [18]. Additionally, the JF-DW samples exhibited similar pH values, independent of the calcium salts used for the treatment.

### 3.3. Nutritional Analyses of Treated R. pulmo Samples

In order to characterize their nutritional values, the JF samples treated by different procedures were analyzed to evaluate their protein content, amino acid composition, antioxidant activity, lipid content, and fatty acids composition. The moisture contents of the different JF samples were: 96.88 ± 1.12 g/100 g FW for the untreated *R. pulmo* JF-DW, 97.85 ± 0.35 g/100 g FW for Ca-Citrate *R. pulmo* JF-DW, 97.6 ± 0.2 g/100 g FW for Ca-Lactate *R. pulmo* JF-DW, 81.94 ± 0.56 g/100 g FW for Salt-Alum *R. pulmo* JF-DW, and 73 ± 0.61 g/100 g FW for Salt-Alum Jp. These data reveal that there were no statistically significant differences existing between the untreated and Ca-Citrate and Ca-Lactate JF-DW *R. pulmo* samples, whereas a substantial reduction in moisture was obtained in both alum-treated JF samples, thus revealing a further difference between the final products obtained by the two types of procedures. The different moisture contents were considered during the analyses and comparisons of nutrient compounds.

#### 3.3.1. Protein Content, Amino Acid Composition, and Antioxidant Activity

**Figure 2:**
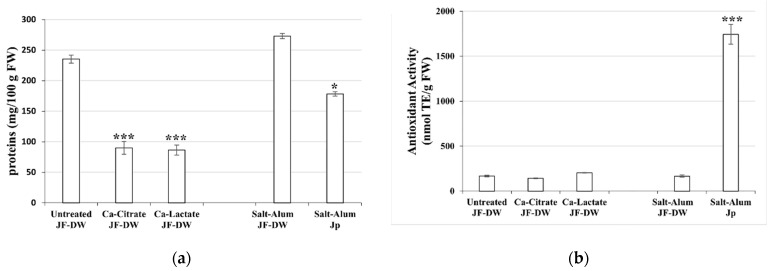
(**a**) Protein content expressed in mg per 100 g of fresh weight (mg/100 g FW) and (**b**) antioxidant activity expressed in nmol TE per gram of fresh weight (nmol TE/g FW) in differently treated samples of *R. pulmo* jellyfish. JF-DW, *R. pulmo* washed with drinking water; Ca-Lactate JF-DW and Ca-Citrate JF-DW, JF samples treated with calcium lactate or calcium citrate brines for 5 days, respectively; Salt-Alum JF-DW, samples of *R. pulmo* JF-DW treated with salt and alum following the traditional method; Salt-alum Jp, commercial ready-to-eat sample of JF produced in Japan by a traditional alum-based method. Values are the means of three independent measurements ± standard deviation. ANOVA statistical test followed by Dunnett’s multiple comparison post hoc test were used to compare each treatment with the control (* *p* < 0.05 and *** *p* < 0.001).

The *R. pulmo* tissues washed with drinking water only (JF-DW) contained 253.2 mg protein per 100 g of fresh weight (FW) (Figure 2a). This value is comparable with the protein content detected by Bleve et al. [18] in JF samples pre-treated with seawater, thus demonstrating that the step of washing with drinking water did not affect the initial protein content. Calcium salt treatment (Ca-Lactate JF-DW and Ca-Citrate JF-DW) significantly reduced the protein content to 60% of the initial value in both samples (88 mg/100 g FW), whereas the traditional salt–alum treatment (Salt-Alum JF-DW) did not affect the protein content (272.9 mg/100 g FW). The commercial ready-to-eat jellyfish sample from Japan (Salt-Alum Jp) contained 178.2 mg protein/100 g FW (Figure 2a), slightly lower than that of the JF-DW and Salt-Alum JF-DW samples, but higher than those of the Ca-Lactate JF-DW and Ca-Citrate JF-DW samples. This evidence suggests that washing with drinking water followed by treatment with calcium salts treatment could lead to a loss of proteins in the processed JF. On the contrary, JF washed with sea water (JF-SW) followed by a 5-day soaking step with Ca-Citrate and Ca-Lactate did not show a significant loss in protein level [18]. This result can be probably explained by a combination of two simultaneous events occurring during the treatments: on one hand, the leakage of solubilized proteinaceous compounds into the brines, and on the other hand, the release of small peptides [30] due to the local denaturation of collagen, being highly susceptible to enzymatic proteolysis [31] under these conditions.

The amino acid composition and content (calculated as the dry weight percentage of lyophilized *R. pulmo* samples) were assayed in untreated JF, JF-DW, and in calcium salts-treated JF (Ca-Citrate JF-DW and Ca-Lactate JF-DW). The total content of amino acids increased from the untreated JF (6%) to the JF washed with drinking water (JF-DW) (9.2%), Ca-Citrate JF-DW (15.4 ± 0.7%), and Ca-Lactate JF-DW (15.3 ± 0.8%) (Table S4). Washing in drinking water and soaking in calcium salt solutions could also cause a leakage of soluble non-proteinaceous components and increase the proteinaceous/amino acid percentage on a dry-weight basis.

The percentages of the taurine, leucine, tyrosine, phenylalanine, and lysine amino acids were higher in the fresh and untreated JF than in the treated JF samples. Increases in the percentages of proline, hydroxyproline, and glycine were also observed in JF-DW and the Ca-treated samples (Ca-Citrate JF-DW and Ca-Lactate JF-DW), with proline and hydroxyproline being abundant in collagen [32], a protein that JF are rich in.

Antioxidant activity (AA) was also evaluated in the same JF samples and expressed in nanomoles of Trolox equivalent per gram of fresh weight (nmol TE/g FW, Figure 2b). Both treatments with either calcium salts or the salt–alum treatment showed similar antioxidant values to those found in JF-DW of approximately 200 nmol TE/g FW. This result indicates that washing the JF with drinking water and successively soaking them in calcium salt-based brines did not affect their antioxidant activity. Furthermore, JF-DW-pre-treated samples showed antioxidant activity levels comparable to those obtained previously by applying the JF-SW procedure [18]. These results could confirm that proteolytic events, possibly due to the treatment, together with the release of small peptides [30], did not affect the final antioxidant activity of the samples. Moreover, the pattern of those released small peptides may be different from those of the low-molecular-weight JF peptides obtained by the controlled enzymatic hydrolysis of jellyfish collagen, as described by De Domenico et al. [25].

It was also observed that the antioxidant activity in the commercial ready-to-eat jellyfish (Salt-Alum Jp) was much higher than that in the treated *R. pulmo* JF-DW (Figure 2b). This evidence could be related to several factors, such as the different JF species used in the commercial product and its high dehydration level, or artificial antioxidants possibly added as preservatives by the manufacturer.

#### 3.3.2. Fatty Acids Composition

In JF-DW, saturated fatty acids (SFAs) accounted for about 50% of total fatty acids (FA), followed by polyunsaturated fatty acids (PUFAs, about 45%) and a small amount of mono-unsaturated fatty acids (MUFAs, 4.3% of the total FA) (Table 3). In *R. pulmo* samples, there was an increase in the SFA percentage, from 50.4% (JF-DW) to 79.3 and 64.4% in Ca-Citrate JF-DW and Ca-Lactate JF-DW, respectively. The SFA content in Salt-Alum JF-DW *R. pulmo* was 81.3% and that in the commercial ready-to-eat jellyfish was 87%. Moreover, Salt-Alum JF-DW and Salt-Alum Jp samples exhibited a more complex lipid profile, since they contained several SFAs that were absent from JF-DW, such as nonadecanoic acid (C19:0), arachidic acid (C20:0), behenic acid (C22:0), and lignoceric acid (C24:0). These differences should be mainly due to the alum treatment of *R. pulmo*. The total MUFA content generally increased in all treated JF samples compared with untreated JF-DW, thus indicating that the initial content of oleic acid (C18:1) was preserved, and also that iso-oleic acid (C18:1 trans-10), palmitoleic acid (C16:1), and vaccenic acid (C18:1 cis-11) appeared.

The increase in the saturated fatty acids content of all treated samples could be due to lipid oxidation, which may lead to isomerization events and the production of new SFA and PUFA species when lipid carbon chains break up and unsaturated FAs are converted to SFAs [33].

The total PUFA content decreased after all the salt treatments. In the Ca-Lactate JF-DW and Ca-Citrate JF-DW samples, the PUFA content decreased to 14.9% and 26%, respectively, from the initial value of 45% in untreated JF-DW. Moreover, PUFAs were heavily reduced in both Salt-Alum JF-DW and Salt-Alum Jp to 4.8 and 4.9%, respectively. However, despite the decrease in quantity, the PUFA composition was still preserved in the calcium-salt-treated JF. Linoleic (C18:2), linolenic (C18:3, ALA), and stearidonic (C18:4) acids were detected in the calcium-treated samples, but not in the JF-DW, while the contents of other nutritionally relevant FAs, such as arachidonic (C20:4), eicosapentaenoic (C20:5, EPA), docosapentaenoic (C22:5, DPA), and docosahexaenoic (C22:6, DHA) acids, were maintained or increased. Interestingly, the novel ω3-PUFA stearidonic acid (C18:4) was detected in the Ca-Lactate JF-DW sample. This FA species is the substrate for the conversion of alpha-linolenic acid (ALA) into longer ω3-PUFAs (EPA, DPA, and DHA) in humans, and it has attracted great interest in recent years because it is obtained only from plants [34].

Overall, in comparison with the corresponding samples obtained by the same authors following the JF-SW method [18], JF-DW pre-treatment in both calcium salt samples exhibited increased values in terms of the total MUFA percentage. This effect was more pronounced for the total PUFA percentages, where increases of 2.3- and 3.3-fold were obtained for JF-DW Ca-citrate and Ca-lactate samples in comparison with the corresponding JF-SW samples [18].

In addition, only the treatments with calcium salts yielded ω6/ω3 ratios less than 1 (0.4 and 0.5 for Ca-Citrate and Ca-Lactate JF-DW, respectively), which represents a healthy composition, as suggested by the nutritional recommendations. The calcium salt-based treatments increased the total lipids concentration in the samples and improved the ratio of essential fatty acids (EFA) naturally present in the untreated material (ω6/ω3 = 2.9). Conversely, the previous not-optimized method proposed by Bleve et al. [18] reported ω6/ω3 ratios of 3.5 and 1.4 for JF-SW Ca-citrate and Ca-lactate treatments, respectively, and 4.6 for JF-DW-Salt-Alum and 4.9 Salt-Alum Jp (traditional salt-alum-based treatment), which are definitely well above the recommended ratio of ω6/ω3 < 1. Since dietary ω3 PUFAs and a balanced ω6/ω3 ratio are needed for the maintenance of human health, the combination of JF-DW and calcium salt treatment proposed here preserved these compounds better than JF-SW and the traditional salt–alum methods. In addition, the total lipid content increased in the samples treated with calcium salts in comparison with the untreated JF-DW (Table 3) and even decreased in the salt–alum-treated samples, thus indicating the protective effects of the calcium salt process on JF lipids, as compared with the traditional method.

#### 3.3.3. Element Content

The profiles of some elements associated with JF samples treated with calcium citrate and calcium lactate brines, as well as with JF treated with salt–alum, revealed the absence or very low levels of cadmium (Cd), lead (Pb) and mercury (Hg) in JF tissues (Table 4). In *R. pulmo* treated with the traditional salt–alum traditional method (Salt-Alum JF-DW), a value of Pb corresponding to 9.748 ppm (or mg/Kg) of dry weight was observed. Notably, the Ca-Citrate JF-DW and Ca-Lactate JF-DW showed lower contents of metals compared with the samples produced with the traditional alum-based process (Salt-Alum JF-DW and Salt-Alum Jp). This evidence was important to demonstrate the unique features of the products obtained by the optimized method described here, since the element composition of the food matrix can directly impact human health and is therefore closely related to food safety.

Chromium (Cr) is considered an essential element, playing a role in the maintenance of carbohydrates, fats, and protein metabolism. However, the levels of this element in Asian-style produced JF should be supervised, since the European suggested daily intake range for humans is 25–200 mg/day [35]. The same considerations can be applied to Vanadium (V), which has a mean dietary intake of about 10–20 µg/person/day or 0.2–0.3 µg/kg body weight/day. Studies in humans revealed gastrointestinal disturbances deriving from the oral intake of vanadium compounds, as well as adverse effects on kidneys and other organs in rats, at relatively low doses. These compounds are not considered essential for humans.

All the tested JF samples did not contain significant levels of Pb, Cd, and Hg, which are considered critical contaminants in foodstuffs [36,37].

Notably, very low levels of aluminum were detected in the Ca-Citrate and Ca-Lactate JF-DW samples. In accordance with other studies [10,38], the data reported here showed very high levels of aluminum in both salt–alum-treated JF, the Salt-Alum JF-DW, and the commercial Salt-Alum Jp, as expected. Regarding the use of alum as a structuring agent for human food, allowed as aluminum sulfates (E 520–523) and sodium aluminum phosphate (E 541), the European Union is very restrictive due to the possible neurotoxic effects of aluminum salts [11], whereas the Joint FAO/WHO Expert Committee on Food Additives (JECFA) set a provisional tolerable weekly intake (PTWI) of 2 mg/kg of body weight [39].

### 3.4. Principal Component Analysis Applied to JF Treated Samples

**Figure 3:**
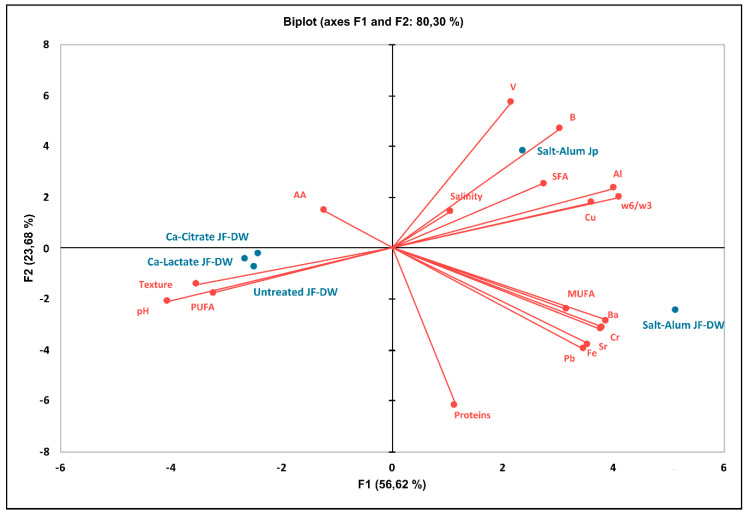
PCA of parameters associated with all treated JF samples. The PCA variables were the data obtained from the analysis of the values of physical traits and the concentrations of chemical compounds at the end of the process. The figure displays the sample scores and variable loadings in the planes formed by PC1–PC2.

A principal component analysis (PCA) was carried out in order to thoroughly compare the JF products obtained by the different treatment methods. The PCA was applied to many relevant parameters, such as the texture, pH, salinity, protein content, fatty acid content, antioxidant activity, and metal content, that can describe the JF samples: JF-DW, Ca-Lactate JF-DW, Ca-Citrate JF-DW, JF treated by the traditional salt–alum method (Salt-Alum JF-DW), and the commercial JF (Salt-Alum Jp) (Figure 3).

A bi-plot is used to show the projection of the variables on the plane defined by the first and second principal components. The total variance of the two main components was 80.3 % (Figure 3). PC1 clustered samples treated with both calcium salts (Ca-Lactate and Ca-Citrate JF-DW) with the untreated JF-DW on the negative semi-axis of the first component, discriminating them from the two salt–alum-treated JF. The clustered group of untreated JF-DW, Ca-Lactate-JF-DW, and Ca-Citrate JF-DW samples were evidently located in the portion of the plane characterized by the pH, texture, antioxidant activity (AA), and PUFA content. The second and the third groups containing Salt-Alum JF-DW and the commercial Salt-Alum Jp, respectively, were located in the opposite portion of the plane, mainly associated with metal ions, particularly aluminum, salinity, SFAs, and MUFAs, and an unfavorable ω6/ω3 ratio.

The application of this unsupervised technique disclosed the considerable difference between the products obtained by the newly proposed method and the traditional methods used to prepare JF for human food uses. The Ca-Lactate-JF-DW and Ca-Citrate JF-DW final products were very close to the fresh untreated JF and were characterized by peculiar food safety and food quality traits.

### 3.5. Application of the New Treatment to Different JF Species

The described JF treatments were also applied to three other putatively edible JF species: *Cotylorhiza tuberculate*, *Rhopilema nomadica*, and *Phyllorhiza punctata*. These three scyphozoa are consumed by humans as food in different areas of the world [1] and were chosen in this study as their presence has also been detected in the Mediterranean Sea.

Food safety and several product features (such as microbiology, texture, pH, and salinity), together with basic nutritional characteristics (such as the fatty acids profile, protein content, and antioxidant activity) were assessed for these samples to evaluate them as potential sea-derived food.

*Safety and quality traits*. The loads of potential pathogenic species, spoilage microbes, and halophilic microbes were not significant in the untreated samples for any of the analyzed JF (Tables S5–S7). After calcium citrate and lactate treatments, the final microbial counts in terms of halophilic species were very low in all three tested JF species, thus suggesting that brine treatment exerts efficient microbial control in all of the different analyzed JF species, despite their distinctive features.

Calcium citrate and calcium lactate treatments improved the texture value of *Cotylorhiza tuberculata* samples by 2.1- and 1.8-fold, respectively. On the other hand, the same treatments led to a decrease in the texture value in the *Rhopilema nomadica* and *Phyllorhiza punctata* samples (Table S8). The latter results are probably related to the non-optimal conditions for storing the samples, having been shipped as frozen material from Israel to Italy and subject to an extended transit time. Therefore, additional tests are planned to confirm the applicability of the proposed procedures to *R. nomadica* and *P. punctata* species.

*Nutritional traits. R. nomadica* and *C. tuberculata* washed with drinking water (JF-DW) showed protein contents of 176.8 mg/100 g FW and 170.3 mg/100 g FW, respectively, (Figure S2a), values that were slightly lower than that measured for *R. pulmo* JF-DW (Figure 2a. On the other hand, *P. punctata* showed a protein content about 2.2-fold higher than that of the other two analyzed species (393.6 mg/100 g FW, Figure S2a). The calcium salt brine treatments decreased the protein content by 60–70% in almost all three JF species, as was previously observed also for *R. pulmo* samples (Figure 2a), with the only exception of *R. nomadica* treated with calcium citrate, where the original value was preserved (Figure S2a).

Additionally, *R. nomadica* treated with calcium citrate kept the same antioxidant activity (AA) value (about 130 nmol TE/g FW, Figure S2b) as that before treatment, whilst the same treatment on *P. punctata* and *C. tuberculata* caused a reduction in AA of about 50% (Ca-Citrate JF-DW, Figure S2b). Furthermore, both *R. nomadica* and *P. punctata* showed a reduction of 40% in the initial AA after calcium lactate treatment, whereas in *C. tuberculate*, the reduction was only about 20%. These data, together with the values obtained for *R. pulmo* (Figure 2), suggested that the calcium brines significantly affected the protein concentration and the choice of calcium salt for the treatment should be adapted to the different JF species.

The FA profiles, reported as the percentage of total FA, were very different between the three JF species (Table S9). *R. nomadica* and *C. tuberculata* JF-DW showed a higher content of SFA than *P. punctata*. PUFAs detected in the JF were probably correlated with the presence of symbiotic species of zooxanthellae microalgae in *C. tuberculata* and *P. punctata*. Calcium salt treatments, mainly calcium citrate, led to a slight increase in the SFA percentage and a reduction in the PUFA content in *R. nomadica* and *C. tuberculata*. In *R. nomadica*, these treatments also led to a reduction in MUFA. On the contrary, *C. tuberculata*, which did not contain any detectable MUFAs in untreated material, contained iso-oleic acid (C18:1 trans-10) and the PUFA isolinolenic acid (C18:2 trans 8,11) when treated with calcium lactate. *P. punctata* treated with calcium salts exhibited a decrease in the SFA content and an increase in the PUFA contents, whereas the levels of MUFAs did not vary compared with the untreated sample. Remarkably, the MUFA iso-oleic acid (C18:1) and PUFA eicosadienoic acid (C20:2) appeared in *P. punctata* Ca-Lactate JF-DW, although they were not initially detectable in the untreated JF sample. However, as shown in Table S9, the essential fatty acids’ (EFAs’) ω-6 to ω-3 ratio in untreated *P. punctata* was less than 1 (ω6/ω3 < 1) and was maintained after the samples were treated with calcium salts (0.4 and 0.3 for Ca-Citrate and Ca-Lactate JFDW, respectively). Among all other treatments, *C. tuberculata* JF-DW treated with calcium citrate was the only species having yielded a favorable ratio of ω6/ω3 < 1 (0.7, Table S9). On the contrary, both *R. nomadica* and *C. tuberculata* samples treated with calcium lactate always achieved a ω6/ω3 ratio higher than the recommended score of 1 (Table S9).

## 4. Conclusions

An optimized method that combines pre-treatment with drinking water followed by a soaking step in calcium salt brine was proposed to stabilize and treat JF for possible food uses in Europe and Western countries. The described procedure for JF-DW pre-treatment improved the fundamental aspects of JF tissue stabilization. The significant reductions in any microbiological growth and undesired enzymatic risks, increased texture values, and desirable antioxidant and fatty acid profiles are some relevant improvements with respect to the very recently proposed JF-SW method [18]. Moreover, the presented approach allowed the content of toxic heavy metals, and especially aluminum, to be strongly reduced. This new, safe approach was initially set up on *R. pulmo* JF species, and later successfully applied to other JF species present in the Mediterranean Sea, thus leading to a preliminary validation of the proposed method. The products obtained by the method described here were used for the formulation of new food prototypes. The characterization of the safety, quality, nutritional, and sensory aspects is ongoing. This study can offer a contribution to fill the knowledge gap in the assessment of JF use as human food in Western countries, even though further important evidence needs to be gathered in terms of toxicological and allergen testing. In addition, the technological simplicity of this process will allow its application in poor coastal environments. As a potential future application, a new commercial kit based on the procedure proposed here could be easily developed and retailed by the same fish shops selling freshly harvested edible JF as a helpful tool for consumers interested in preparing homemade and safe JF-based dishes.

## 5. Patents

The optimized method for JF treatment and stabilization described in this manuscript is part of the European Patent EP 3763224, deposited on 2020 [40].

## Figures and Tables

**Table 1:** Safety and quality parameters applied to JF-B (JF directly pre-treated on the boat) and JF-DW (JF washed with drinking water). Samples were treated for 5 days with calcium citrate (Ca-Citrate) or calcium lactate (Ca-Lactate) brines, following accredited conventional assays used for seafood and fish-derived products (as already described by Bleve et al. [15]). The different letters in line indicate significant differences between samples (*p* < 0.05).

Accredited Analysis	Ca-Citrate		Ca-Lactate	
	JF-B	JF-DW	JF-B	JF-DW
	CFU/g	CFU/g	CFU/g	CFU/g
**Total bacteria**	<10 (a)	1.30 × 10^3^ ± 1.23 × 10^1^ (b)	<10 (a)	3.70 × 10^2^ ± 2.31 (c)
**Coliforms**	<10 (a)	<10 (a)	<10 (a)	<10 (a)
***Escherichia coli***	<10 (a)	<10 (a)	<10 (a)	<10 (a)
**Staphylococci**	1.60 × 10^2^ ± 5.21 (a)	1.00 x 10^2^ ± 7.25 (a)	1.00 × 10^2^ ± 8.32 (a)	7.30 × 10^1^ ± 6.15 (a)
**Yeast and Molds**	<10 (a)	<10(a)	<10 (a)	<10 (a)
	**presence/25 g**	**presence/25 g**	**presence/25 g**	**presence/25 g**
***Salmonella* spp.**	0 (a)	0 (a)	0 (a)	0 (a)
***Listeria monocytogenes***	0 (a)	0 (a)	0 (a)	0 (a)
	**mg/Kg**	**mg/Kg**	**mg/Kg**	**mg/Kg**
**Histamine**	<3 (a)	<3 (a)	<3 (a)	<3 (a)
	**mg/100 g**	**mg/100 g**	**mg/100 g**	**mg/100 g**
**TBVN**	<0.1 (a)	<0.1 (a)	2.5 ± 0.1 (b)	<0.1 (a)

**Table 2:** Texture, pH, and salinity values of *R. pulmo* JF-B (JF directly pre-treated on the boat) and JF-DW (JF washed with drinking water). JF samples were untreated and treated with brines containing different calcium salts at 5 days of treatment (Ca-Citrate: calcium citrate; Ca-Lactate: calcium lactate), and JF-DW was treated with salt–alum (obtained after 20 days at 4 °C and 2 days of air drying, as described in the Material and Methods section).

Pre-Treatments	Treatments			
	Untreated	Ca-Citrate	Ca-Lactate	Salt-Alum
	**Texture (N)**			
**JF-B**	−74 ± 15 (a)	−34 ± 11 (b)	−53 ± 8 (b)	n.d.
**JF-DW**	−75 ± 10 (a)	−137 ± 15 (b)	−117 ± 5 (b)	−43 ± 11(c)
	**Salinity (%)**			
**JF-B**	3.5 ± 0.1 (a)	2 ± 0.2 (b)	2.4 ± 0.1 (b)	n.d.
**JF-DW**	3.5 ± 0.2 (a)	1.5 ± 0.3 (b)	1.8 ± 0.2 (b)	2.5 ± 0.1 (c)
	**pH**			
**JF-B**	7.1 ± 0.2 (a)	4.72 ± 0.2 (b)	7.06 ± 0.3 (a)	n.d.
**JF-DW**	6.9 ± 0.3 (a)	5.2 ± 0.1 (b)	5.56 ± 0.4 (b)	3.65 ± 0.4 (c)

The different letters in line indicate significant differences between the samples (*p* < 0.05). N: Newton; n.d., not determined.

**Table 3:** Fatty acid composition of *R. pulmo* JF samples. They were washed with drinking water (JF-DW), treated with brines containing calcium salts (Ca-Citrate JF-DW and Ca-Lactate JF-DW), or treated with the salt–alum method (Salt-Alum JF-DW); a commercial JF sample from Japan treated by the salt–alum method (Salt-Alum Jp) was also tested. Fatty acid composition data are expressed as the percentage of the total fatty acids ± SD.

Fatty Acids Composition (%)					
	*Rhizostoma Pulmo* Samples				Commercial JF
	JF-DW	Ca-Citrate JF-DW	Ca-Lactate JF-DW	Salt-Alum JF-DW	Salt-Alum Jp
**Saturated FA (SFA)**					
**Myristic acid C14:0**	4.0 ± 0.4	7.5 ± 0.8	7.2 ± 0.7	4.1 ± 0.4	2.6 ± 0.3
**Pentadecanoic acid C15:0**	––	––	––	1.7 ± 0.2	––
**Palmitic acid C16:0**	23.5 ± 2.5	34.9 ± 4.3	28.4 ± 0.3	35.4 ± 3.5	31.5 ± 3.1
**Margaric acid C17:0**	1.1 ± 0.2	1.3 ± 0.3	3.6 ± 0.1	4.8 ± 0.5	2.5 ± 0.3
**Stearic acid C18:0**	21.8 ± 2.1	35.6 ± 4.2	22.4 ± 2.3	32.9 ± 3.3	47.2 ± 4.8
**Nonadecanoic acid C19:0**	––	––	2.8 ± 0.3	0.7 ± 0.1	0.6 ± 0.1
**Arachidic acid C20:0**	––	––	––	1.1 ± 0.1	1.5 ± 0.1
**Behenic acid C22:0**	––	––	––	0.3 ± 0.1	0.7 ± 0.1
**Lignoceric acid C24:0**	––	––	––	0.3 ± 0.1	0.6 ± 0.1
**Total SFA**	50.4 ± 5.1	79.3 ± 0.8	64.4 ± 6.5	81.3 ± 8.2	87.1 ± 8.7
**Monounsaturated FA (MUFA)**					
**Palmitoleic acid C16:1 (ω7)**	––	2.7± 0.3	4.5± 0.5	3.5 ± 0.4	2.9 ± 0.3
**Oleic acid C18:1 (ω9)**	2.5 ± 2.1	2.0 ± 0.2	3.1 ± 0.3	1.4 ± 0.1	––
**Iso-oleic acid C18:1 trans-10**	1.8 ± 0.2	––	2.0 ± 0.2	––	1.3 ± 0.1
**Vaccenic acid C18:1 cis-11 (ω7)**	––	1.1 ± 0.1	––	5.3 ± 0.5	3.8 ± 0.4
**Trans-vaccenic acid C18:1 trans-13**	––	––	––	2.8 ± 0.3	––
**Paullinic acid C20:1 (ω7)**	––	––	––	0.5 ± 0.1	––
**Total MUFA**	4.3 ± 0.5	5.8 ± 0.6	9.6 ± 0.1	13.9 ± 1.4	8.0 ± 0.8
**Polyunsaturated FA (PUFA)**					
**Linoleic acid C18:2 (ω6)**	––	––	3.1 ± 0.5	2.0 ± 0.2	3.9 ± 0.4
**Isolinoleic acid C18:2 trans 8.11**	––	3.9 ± 0.4	––	––	––
**Linolenic acid C18:3 (ω3)**	––	––	2.0 ± 0.2	––	––
**Stearidonic acid C18:4 (ω3)**	––	––	2.8 ± 0.3	––	––
**Eicosadienoic acid C20:2 (ω6)**	––	––	––	––	––
**Arachidonic acid C20:4 (ω6)**	33.8 ± 3.4	3.6 ± 0.4	4.5 ± 0.1	2.0 ± 0.2	1.0 ± 0.1
**Eicosapentaenoic acid C20:5 (ω3)**	5.4 ± 0.4	4.0 ± 0.4	6.3 ± 0.6	0.4 ± 0.1	––
**Docosapentaenoic acid C22:5 (ω3)**	2.1 ± 0.2	––	1.0 ± 0.1	––	––
**Docosahexaenoic acid C22:6 (ω3)**	4.1 ± 0.4	3.4 ± 0.3	6.3 ± 0.6	0.4 ± 0.1	––
**Total PUFA**	45.4 ± 4.5	14.9 ± 1.5	26.0 ± 1.9	4.8 ± 0.5	4.9 ± 0.5
**Σω6**	33.8 ± 3.4	3.6 ± 0.4	7.6 ± 0.6	4.0 ± 0.4	4.9 ± 0.5
**Σω3**	11.6 ± 1.0	7.4 ± 0.7	18.4 ± 1.8	0.9 ± 0.2	––
**ω6/ω3**	2.9	0.5	0.4	4.6	4.9
**Total Lipids (%DW)**	8.3 ± 0.9	13.2 ± 1.2	12.5 ± 1.3	6.3 ± 0.5	3.6 ± 0.4

**Table 4:** Elements evaluated in the samples of *R. pulmo* subjected to different treatments. JF-DW, JF samples washed with drinking water; Ca-Citrate JF-DW and Ca-Lactate JF-DW, *R. pulmo* JF treated with brines containing calcium citrate or calcium lactate, respectively; Salt-Alum JF-DW, JF samples treated with an alum-based procedure; Salt-Alum Jp, commercial JF treated with alum from Japan. Data are expressed in ppm ± SD; the different letters in line indicate significant differences between samples (*p* < 0.05).

	*Rhizostoma Pulmo*			Commercial
Elements	Ca-Citrate JF-DW	Ca-Lactate JF-DW	Salt-Alum JF-DW	Salt-Alum Jp
Ppm				
**Ag**	0 (a)	0 (a)	8.554 ± 0.432 (b)	9.844 ± 0.432 (c)
**Al**	0.05722 ± 0.00411 (a)	0.20558 ± 0.04742 (a)	5213.552 ± 157.573 (b)	5979.045 ± 104.524 (c)
**As**	0.02667 ± 0.00103 (a)	0.03526 ± 0.00016 (a)	0.202 ± 0.002 (b)	0.236 ± 0.019 (c)
**B**	0.71483 ± 0.13397 (a)	0.71211 ± 0.16684 (a)	33.981 ± 0.255 (b)	84.628 ± 0.838 (c)
**Ba**	0.02983 ± 0.00486 (a)	0.02834 ± 0.00413 (a)	44.510 ± 0.289 (b)	8.693± 0.350 (c)
**Bi**	0	0	0	0
**Ca**	1480.38 ± 51.1962 (b)	736.053 ± 16.5789 (a)	632.589± 0.178 (c)	141.498 ± 0.090 (d)
**Cd**	0.0016 ± 0.00036 (a)	0.00163 ± 0.00021 (a)	0 (b)	0 (b)
**Co**	0.00055 ± 0.00008 (b)	0.00071 ± 0.00008 (a)	0 (c)	0 (c)
**Cr**	0.00072 ± 0.00005 (a)	0.00226 ±0.00009 (a)	87.544 ± 0.187 (b)	11.906 ± 0.131 (c)
**Cu**	0.02392 ± 0.00483 (a)	0.02484 ± 0.00563 (a)	8.180 ± 0.037 (b)	8.578 ± 0.124 (c)
**Fe**	0.09768 ± 0.04256 (a)	0.18979 ± 0.01947 (a)	284.792 ± 8.383 (b)	10.189 ± 1.345 (c)
**Hg**	0 (b)	0.01079 ± 0.00789 (a)	0 (b)	0 (b)
**In**	0	0	0	0
**K**	116.364 ± 29.4737 (a)	121.895 ± 40.5789 (a)	12021.737± 135.355 (b)	1241.373 ± 18.463(c)
**Li**	0.02682 ± 0.00457 (a)	0.02668 ± 0.00526 (a)	1.145 ± 0.008 (b)	0.536 ± 0.018 (c)
**Mg**	642.584 ± 91.866 (a)	545.632 ± 156.474 (a)	1664.770 ± 62.521 (b)	352.398 ± 9.879 (a,d)
**Mn**	0.00653 ± 0.00022 (a)	0.02137 ± 0.00153 (a)	140.681 ± 0.071 (b)	3.532 ± 0.203 (c)
**Mo**	0.0028 ± 0.00008 (a)	0.00339 ± 0.00008 (a)	2.084 ± 0.100 (b)	5.286 ± 0.551 (c)
**Na**	3858.61 ± 715.55 (a)	3165.26 ± 1127.89 (a)	>10000 (b)	>10000 (b)
**Ni**	0.00069 ± 0.00017 (b)	0.00147 ± 0.00011 (a)	0 (c)	0 (c)
**Pb**	0 (a)	0 (a)	9.748 ± 0.372 (b)	0 (a)
**Sr**	2.88278 ± 0.47129 (a)	2.77105 ± 0.48684 (a)	25.813 ± 9.1158 (b)	5.960 ± 0.157 (a,b)
**Te**	0.00012 ± 0.0002 (b)	0.00103 ± 0.0005 (a)	0 (b,c)	0 (b,c)
**Tl**	0	0	0	0
**V**	0.47493 ± 0.08632 (a)	0.46234 ± 0.10503 (a)	12.180 ± 0.034 (b)	81.073 ± 0.002 (b)
**Zn**	0.34144 ± 0.03876 (a)	0.47313 ± 0.02434 (a)	36.019 ± 0.221 (b)	2.606 ± 0.033 (b)

## Data Availability

All related data and methods are presented in this paper. Additional inquiries should be addressed to the corresponding author.

## Supplementary Materials

The following supporting information can be downloaded at: https://www.mdpi.com/article/10.3390/foods11172697/s1, Figure S1. *Rhizostoma pulmo* jellyfish products obtained by the new proposed method. Ca-Citrate JF-DW: JF after treatment with calcium citrate brine; Ca-Lactate JF-DW: JF after treatment with calcium lactate brine; Salt-Alum JF-DW: JF obtained by the salt–alum-based traditional method. Figure S2. Proteins content (a) and Antioxidant activity (b) in *Cotylorhiza tuberculata*, *Rhopilema nomadica* and *Phyllorhiza punctata* JF-DW samples and the corresponding calcium salt treated samples (Ca-Lactate JF-DW and Ca-Citrate JF-DW). Protein contents were expressed as mg per 100 g of fresh weight (mg/100 g FW) and antioxidant activity was expressed as nmol TE per gram of FW (nmol TE/g FW). Values are the means of three independent measurements, ±standard deviation. ANOVA statistic test followed by Bonferroni’s multiple comparison post-hoc test was used to compare each treatment with the others for each JF species. Table S1. Microbiological analyses of JF-DW (JF transported on lab and pre-treated with drinking water) untreated and treated with brines containing different calcium salts at 5 days treatment at 4 °C (Ca-Citrate: calcium citrate; Ca-Lactate: calcium lactate). The different letters in line indicate significant differences between samples (*p* < 0.05). TBC: total bacterial count at 30 °C; sCMA: saline Corn Meal Agar; sSDA: saline Sabouraud Dextrose Agar. Table S2. Microbiological analyses of JF-B (JF directly pre-treated on boat) untreated and treated with brines containing different calcium salts at 5 days treatment at 4 °C (Ca-Citrate: calcium citrate; Ca-Lactate: calcium lactate). The different letters in line indicate significant differences between samples (*p* < 0.05). TBC: total bacterial count at 30 °C; sCMA: saline Corn Meal Agar; sSDA: saline Sabouraud Dextrose Agar. Table S3. Microbiological analyses of *Rhizostoma pulmo* JF sample washed in drinking water and then treated with NaCl-alum (Salt-Alum JF-DW). This sample was obtained after 20 days at 4 °C and 2 days air drying (as described in Material and Methods section). TBC: total bacterial count at 30 °C; sCMA: saline Corn Meal Agar; sSDA: saline Sabouraud Dextrose Agar. Table S4. Amino acid composition of *Rhizostoma pulmo* JF fresh and untreated (Untreated JF), washed in drinking water (JF-DW), and treated with calcium citrate (Ca-Citrate JF-DW) and calcium lactate (Ca-Lactate JF-DW). Data are mean ± Standard deviation (±SD) of four independent analyses. AA. amino acids; Nd. Not detected. Table S5. Microbiological analyses of *Cotylorhiza tuberculata* JF sample washed with drinking water (JF-DW) and treated with brines containing calcium salts (Ca-Citrate: calcium citrate; Ca-Lactate: calcium lactate). The different letters in line indicate significant differences between samples (*p* < 0.05). TBC: total bacterial count at 30 °C; sCMA: saline Corn Meal Agar; sSDA: saline Sabouraud Dextrose Agar. Table S6. Microbiological analyses of *Rhopilema nomadica* JF sample washed with drinking water (JF-DW) and treated with brines containing calcium salts (Ca-Citrate: calcium citrate; Ca-Lactate: calcium lactate). The different letters in line indicate significant differences between samples (*p* < 0.05). TBC: total bacterial count at 30 °C; sCMA: saline Corn Meal Agar; sSDA: saline Sabouraud Dextrose Agar. Table S7. Microbiological analyses of *Phyllorhiza punctata* JF sample washed with drinking water (JF-DW) and treated with brines containing calcium salts (Ca-Citrate: calcium citrate; Ca-Lactate: calcium lactate). The different letters in line indicate significant differences between samples (*p* < 0.05). TBC: total bacterial count at 30 °C; sCMA: saline Corn Meal Agar; sSDA: saline Sabouraud Dextrose Agar. Table S8. Texture, salinity and pH values of *Cotylorhiza tuberculata*. *Rhopilema nomadica* and *Phyllorhiza punctata* JF samples washed with drinking water (JF-DW) and treated with brines containing calcium salts at 5 days treatment (Ca-Citrate: calcium citrate; Ca-Lactate: calcium lactate). The different letters in line indicate significant differences between samples (*p* < 0.05). Table S9. Comparison of the fatty acid composition of and *Rhopilema nomadica*. *Phylloriza punctata* and *Cothyloriza tuberculata* JF samples washed with drinking water (JF-DW) and treated with brines containing calcium salts at 5 days treatment (Ca-Citrate: calcium citrate; Ca-Lactate: calcium lactate). Fatty acid composition data are expressed as percentage of the total fatty acids ± SD.
