# Supplementary material for: Optimization of a Calcium-Based Treatment Method for Jellyfish to Design Food for the Future
Source: Foods. 2022 Sep 4;11(17):2697. doi: 10.3390/foods11172697 (PMC9455609; doi:10.3390/foods11172697)
Supplement: Supplementary file 1 [file foods-11-02697-s001.zip › foods-1848637-supplementary.pdf]

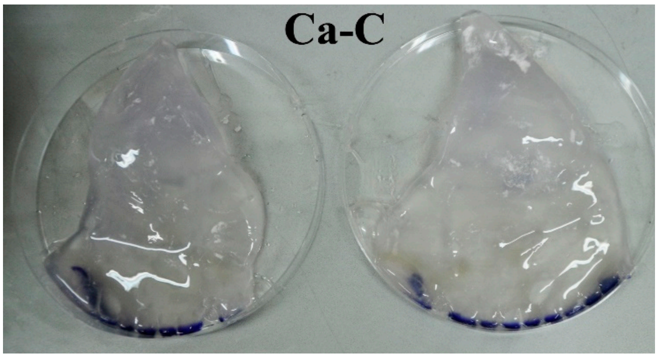

(**a**)

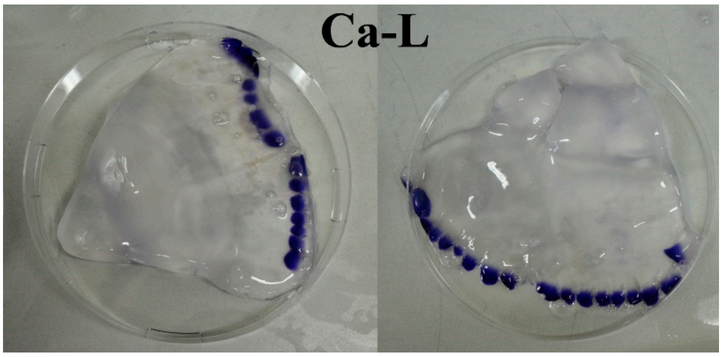

(**b**)

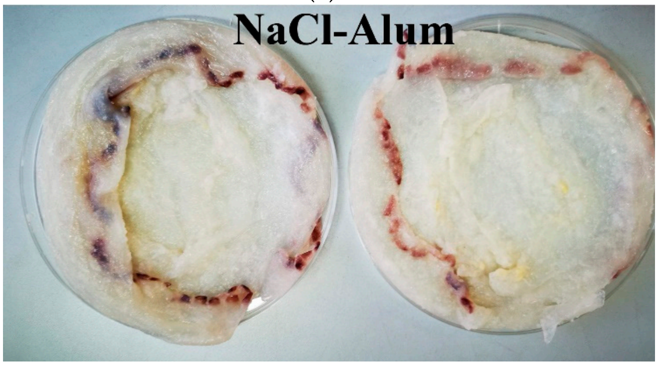

(c)

Figure S1. *Rhizostoma pulmo* jellyfish products obtained by the new proposed method. Ca-C: *R. pulmo* jellyfish after treatment in calcium citrate brine (a); Ca-L: *R. pulmo* jellyfish after treatment in calcium lactate brine (b); NaCl-Alum: *R. pulmo* jellyfish obtained by salt-alum traditional method (c).

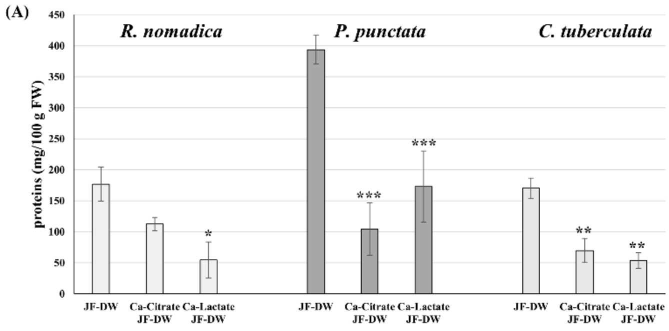

(**a**)

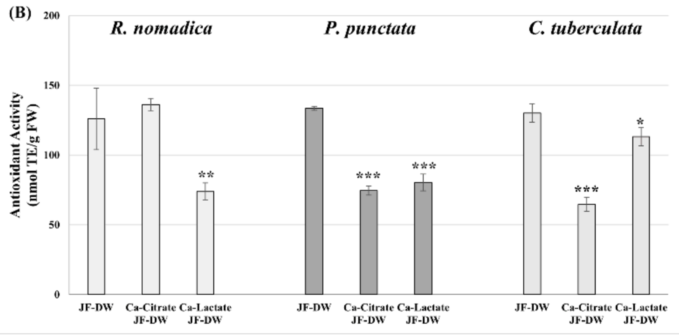

(**b**)

Figure S2. Proteins content (a) and Antioxidant activity (b) in *Cotylorhiza tuberculata, Rhopilema nomadica* and *Phyllorhiza punctata* JF-DW samples and the corresponding calcium salt treated samples (Ca-Lactate JF-DW and Ca-Citrate JF-DW). Protein contents were expressed as mg per 100 grams of fresh weight (mg/100 g FW) and antioxidant activity was expressed as nmol TE per gram of FW (nmol TE/g FW). Values are the means of three independent measurements, ± standard deviation. ANOVA statistic test followed by Bonferroni's multiple comparison post-hoc test was used to compare each treatment with the others for each JF species.

Table S1. Microbiological analyses of JF-DW (JF transported on lab and pre-treated with drinking water) untreated and treated with brines containing different calcium salts at 5 days treatment at 4 °C (Ca-Citrate: calcium citrate; Ca-Lactate: calcium lactate). The different letters in line indicate significant differences between samples ($p < 0.05$)

Microorganisms	Medium	JF-DW					
		Untreated		Ca-Citrate		Ca-Lactate	
		Mean (CFU/g)	SD	Mean (CFU/g)	SD	Mean (CFU/g)	SD
**TBC**	**PCA**	0 (a)	0	8.75 x 10^1 (b)	3.54	2.90 x 10^2 (c)	5.66 x 10^1
***Bacillus* spp.**	**BCSA**	3.00 (a)	1.05	0 (b)	0	0 (b)	0
**H_2S-producing bacteria**	**IRON AGAR**	0	0	0	0	0	0
**Enterobacteriaceae**	**VRBGA**	0	0	0	0	0	0
***Coli-Aerogenes* Bacteria**	**VRBA**	0	0	0	0	0	0
**Coagulase positive staphylococci**	**Baird Parker Agar**	0	0	0	0	0	0
**Pathogenic staphylococci**	**MSA**	0	0	0	0	0	0
***Pseudomonas* spp.**	**Pseudomonas Agar**	0	0	0	0	0	0
***Vibrio* spp.**	**TCBSA**	0	0	0	0	0	0
**Yeasts**	**DRBC**	0	0	0	0	0	0
**Moulds**		0	0	0	0	0	0
**Halophilic Bacteria**	**R2A**	0 (a)	0	1.15 x 10^1 (a)	2.12	6.75 x 10^1 (b)	3.54
	**Marine Agar**	0	0	0	0	0	0
**Halophilic Yeasts**	**sCMA**	0	0	0	0	0	0
	**sSDA**	0	0	0	0	0	0
	**R2A**	0	0	0	0	0	0
	**Marine Agar**	0	0	0	0	0	0
**Halophilic Moulds**	**sCMA**	0	0	0	0	0	0
	**sSDA**	0	0	0	0	0	0
	**R2A**	0	0	0	0	0	0
	**Marine Agar**	0	0	0	0	0	0

TBC: total bacterial count at 30 °C; sCMA: saline Corn Meal Agar; sSDA: saline Sabouraud Dextrose Agar

Table S2. Microbiological analyses of JF-B (JF directly pre-treated on boat) untreated and treated with brines containing different calcium salts at 5 days treatment at 4 °C (Ca-Citrate: calcium citrate; Ca-Lactate: calcium lactate). The different letters in line indicate significant differences between samples ($p < 0.05$)

Microorganisms	Medium	JF-B					
		Untreated		Ca-Citrate		Ca-Lactate	
		Mean (CFU/g)	SD	Mean (CFU/g)	SD	Mean (CFU/g)	SD
**TBC**	**PCA**	4.60 x 10^1 (a)	4.58	7.50(b)	2.12	6.40 x 10^1 (a)	1.98 x 10^1
***Bacillus* spp.**	**BCSA**	2.40 x 10^1 (a)	5.66	0 (b)	0	1.35 x 10^1 (a)	2.12
**H_2S-producing bacteria**	**IRON AGAR**	0	0	0	0	0	0
**Enterobacteriaceae**	**VRBGA**	0 (a)	0	0 (a)	0	1.45 x 10^1 (b)	8.15 x 10^{-1}
***Coli-Aerogenes* Bacteria**	**VRBA**	0	0	0	0	0	0
**Coagulase positive staphylococci**	**Baird Parker Agar**	0	0	0	0	0	0

**Pathogenic staphylococci**	**MSA**	0	0	0	0	0	0
***Pseudomonas* spp.**	**Pseudomonas Agar**	3.50 (a)	5.08 x 10^1	0 (b)	0	0 (b)	0
***Vibrio* spp.**	**TCBSA**	0	0	0	0	0	0
**Yeasts**	**DRBC**	0	0	0	0	0	0
**Moulds**		0	0	0	0	0	0
**Halophilic Bacteria**	**R2A**	7.60 x 10^1 (a)	7.16	0 (b)	0	0 (b)	0
	**Marin Agar**	0	0	0	0	0	0
**Halophilic Yeasts**	**sCMA**	0	0	0	0	0	0
	**sSDA**	0	0	0	0	0	0
	**R2A**	0 (a)	0	0 (a)	0	2.75 x 10^1 (b)	1.06 x 10^1
	**Marine Agar**	0	0	0	0	0	0
**Halophilic Moulds**	**sCMA**	0	0	0	0	0	0
	**sSDA**	0	0	0	0	0	0
	**R2A**	0	0	0	0	0	0
	**Marine Agar**	0	0	0	0	0	0

TBC: total bacterial count at 30 °C; sCMA: saline Corn Meal Agar; sSDA: saline Sabouraud Dextrose Agar

Table S3. Microbiological analyses of *Rhizostoma pulmo* JF sample washed in drinking water and then treated with NaCl-alum (Salt-Alum JF-DW). This sample was obtained after 20 days at 4 °C and 2 days air drying (as described in Material and Methods section).

Microorganisms	Medium	Mean (CFU/g)	SD
**TBC**	**PCA**	5.80x 10^1	3.11x10^1
***Bacillus* spp.**	**BCSA**	4.15x10^1	1.20x10^1
**H_2S-producing bacteria**	**IRON AGAR**	0	0
**Enterobacteriaceae**	**VRBGA**	0	0
***Coli-Aerogenes* Bacteria**	**VRBA**	0	0
**Coagulase positive staphylococci**	**Baird Parker Agar**	0	0
**Pathogenic staphylococci**	**MSA**	0	0
***Pseudomonas* spp.**	**Pseudomonas Agar**	0	0
***Vibrio* spp.**	**TCBSA**	0	0
**Yeasts**	**DRBC**	1.30x10^2	1.41x10^1
**Moulds**		0	0
**Halophilic Bacteria**	**R2A**	9.88x10^2	2.23x10^2
	**Marine Agar**	4.43x10^2	2.49x10^2
**Halophilic Yeasts**	**sCMA**	2.22x10^2	8.20x10^1
	**sSDA**	5.00x10^2	4.24x10^2
	**R2A**	1.16x10^3	6.22x10^2
	**Marine Agar**	9.00x10^2	4.24x10^2
**Halophilic Moulds**	**sCMA**	0	0
	**sSDA**	0	0
	**R2A**	0	0
	**Marine Agar**	0	0

TBC: total bacterial count at 30° C; sCMA: saline Corn Meal Agar; sSDA: saline Sabouraud Dextrose Agar

foods MDPI

Table S4. Amino acid composition of *Rhizostoma pulmo* JF fresh and untreated (Untreated JF)), washed in drinking water (JF-DW), and treated with calcium citrate (Ca-Citrate JF-DW) and calcium lactate (Ca-Lactate JF-DW). Data are mean ± Standard deviation (± SD) of four independent analyses. AA. amino acids; Nd. Not detected.

	Rhizostoma pulmo samples							
	Untreated JF		JF-DW		Ca-Citrate JF-DW		Ca-Lactate JF-DW	
**Amino acids**	Percentage per dry weight (% of dw) Average ± SD	Percentage of total AA (%)	Percentage per dry weight (% of dw) Average ± SD	Percentage of total AA (%)	Percentage per dry weight (% of dw) Average ± SD	Percentage of total AA (%)	Percentage per dry weight (% of dw) Average ± SD	Percentage of total AA (%)
**Taurin**	0.3 ± 0.1	5.4	0.1 ± 0.1	0.6	0.1 ± 0.1	0.9	0.2 ± 0.1	1.2
**Methionine sulfoxide**	n.d.	-	n.d.	-	n.d.	-	n.d.	-
**Hydroxyproline**	0.0	-	0.1 ± 0.1	1.2	0.2 ± 0.2	1.3	0.2 ± 0.2	1.1
**Aspartic acid + Asparagine**	0.4 ± 0.1	5.9	0.6 ± 0.2	6.8	1.0 ± 0.1	6.7	1.0 ± 0.2	6.5
**Threonine**	0.2 ± 0.0	3.6	0.3 ± 0.1	3.4	0.6 ± 0.0	4.2	0.7 ± 0.1	4.7
**Serine**	0.3 ± 0.0	5.3	0.5 ± 0.1	5.0	0.9 ± 0.2	6.0	0.8 ± 0.2	5.6
**Glutamic acid + Glutamine**	0.6 ±0.1	9.9	0.9 ± 0.3	9.4	1.7 ± 0.1	11.1	1.6 ± 0.1	10.4
**Proline**	0.2 ±0.2	3.0	0.5 ± 0.3	5.5	1.1 ± 0.2	7.2	1.1 ± 0.1	7.4
**Glycine**	0.6 ± 0.0	9.4	1.6 ± 0.4	17.0	2.4 ± 0.0	15.5	2.2 ± 0.1	14.4
**Alanine**	0.4 ±0.0	7.0	0.8 ± 0.2	8.5	1.1 ± 0.2	7.2	1.5 ± 0.3	9.5
**Cystine (Cys-Cys)**	0.0 ± 0.0	0.5	n.d.	-	0.0 ± 0.1	0.3	0.1 ± 0.1	0.4
**Valine**	0.3 ± 0.0	5.0	0.4 ± 0.1	4.0	0.8 ± 0.0	5.1	0.8 ± 0.0	5.3
**Methionine**	0.0 ± 0.0	0.8	0.1 ± 0.1	0.6	0.2 ± 0.0	1.3	0.2 ± 0.0	1.3
**Isoleucine**	0.2 ± 0.0	3.8	0.3 ± 0.1	3.2	0.5 ± 0.0	3.3	0.5 ± 0.0	3.0
**Leucine**	0.3 ± 0.0	5.8	0.4 ± 0.1	4.5	0.7 ± 0.0	4.6	0.7 ± 0.0	4.4
**Tyrosine**	0.3 ± 0.1	5.8	0.3 ± 0.1	3.7	0.5 ± 0.1	3.3	0.4 ± 0.1	2.9
**Phenylalanine**	0.4 ± 0.1	7.4	0.4 ± 0.1	4.8	0.7 ± 0.1	4.5	0.7 ± 0.2	4.5
**Histidine**	0.2 ± 0.0	2.9	0.3 ± 0.1	3.5	0.4 ± 0.0	2.9	0.5 ± 0.1	2.9
**Hydroxylysine**	n.d.	-	n.d.	-	n.d.	-	n.d.	-
**Lysine**	0.4 ± 0.0	7.3	0.5 ± 0.1	5.5	0.8 ± 0.0	5.1	0.8 ± 0.0	5.1
**Tryptophan**	n.d.		n.d.	-	n.d.	-	n.d.	-

**Arginine**	0.7 ± 0.2	11.1	1.2 ± 0.4	12.7	1.5 ± 0.4	9.8	1.4 ± 0.3	9.2
Ammonia	0.1 ± 0.0	1.7	0.1 ± 0.0	1.5	0.2 ± 0.0	1.6	0.2 ± 0.0	1.5
**Total (AA only)**	6.0 ± 0.1	**100.0**	**9.2 ± 2.1**	**100.0**	**15.4 ± 0.7**	**100.0**	**15.3 ± 0.8**	**100.0**
Total (AA + ammonia)	6.1 ± 0.1		9.3 ± 2.1		15.7 ± 0.7		15.5 ± 0.8	

Table S5. Microbiological analyses of *Cotylorhiza tuberculata* JF sample washed with drinking water (JF-DW) and treated with brines containing calcium salts (Ca-Citrate: calcium citrate; Ca-Lactate: calcium lactate). The different letters in line indicate significant differences between samples ($p < 0.05$)

		Cotylorhiza tuberculata					
**Microorganisms**	**Medium**	**JF-DW**		**Ca-Citrate**		**Ca-Lactate**	
		**Mean (CFU/g)**	**SD**	**Mean (CFU/g)**	**SD**	**Mean (CFU/g)**	**SD**
**TBC**	**PCA**	0	0	0	0	0	0
***Bacillus* spp.**	**BCSA**	0	0	0	0	0	0
**H_2S-producing bacteria**	**IRON AGAR**	0	0	0	0	0	0
**Enterobacteriaceae**	**VRBGA**	0	0	0	0	0	0
***Coli-Aerogenes* Bacteria**	**VRBA**	0	0	0	0	0	0
**Coagulase positive staphylococci**	**Baird Parker Agar**	0	0	0	0	0	0
***Pseudomonas* spp.**	**Pseudomonas Agar**	0	0	0	0	0	0
**Yeasts**	**DRBC**	0	0	0	0	0	0
**Moulds**		0 (a)	0	0 (a)	0	3.60x10^1(b)	1.69 x10^1
**Halophilic Bacteria**	**R2A**	0 (a)	0	2.80x 10^1 (b)	8.48	1.60x10^1(b)	1.13 x10^1
	**Marine Agar**	0 (a)	0	1.20 x10^1 (a)	2.82	3.21 x10^1 (b)	1.56 x10^1
**Halophilic Yeasts**	**sCMA**	0 (a)	0	0 (a)	0	2.53 x10^1(b)	1.75 x10^1
	**sSDA**	0 (a)	0	0 (a)	0	3.20x 10^1(b)	1.18 x10^1
	**R2A**	0 (a)	0	0 (a)	0	1.20 x10^1(b)	5.65
	**Marine Agar**	0	0	0	0	0	0
**Halophilic Moulds**	**sCMA**	0	0	0	0	0	0
	**sSDA**	0	0	0	0	0	0
	**R2A**	0	0	0	0	0	0
	**Marine Agar**	0	0	0	0	0	0

TBC: total bacterial count at 30°C; sCMA: saline Corn Meal Agar; sSDA: saline Sabouraud Dextrose Agar

Table S6. Microbiological analyses of *Rhopilema nomadica* JF sample washed with drinking water (JF-DW) and treated with brines containing calcium salts (Ca-Citrate: calcium citrate; Ca-Lactate: calcium lactate). The different letters in line indicate significant differences between samples ($p < 0.05$)

Rhopilema nomadica							
**Microorganisms**	**Medium**	**JF-DW**		**Ca-Citrate**		**Ca-Lactate**	
		**Mean (CFU/g)**	**SD**	**Mean (CFU/g)**	**SD**	**Mean (CFU/g)**	**SD**
**TBC**	**PCA**	4.20×10^1 (a)	1.27×10^1	3.60×10^1 (a)	5.74	0 (b)	0
***Bacillus* spp.**	**BCSA**	0 (a)	0	2.40×10^1 (b)	7.48	0 (a)	0
**H_2S-producing bacteria**	**IRON AGAR**	0	0	0	0	0	0
**Enterobacteriaceae**	**VRBGA**	0	0	0	0	0	0
***Coli-Aerogenes* Bacteria**	**VRBA**	0	0	0	0	0	0
**Coagulase positive staphylococci**	**Baird Parker Agar**	0	0	0	0	0	0
***Pseudomonas* spp.**	**Pseudomonas Agar**	0	0	0	0	0	0
**Yeasts**	**DRBC**	0	0	0	0	0	0
**Moulds**		2.40×10^1 (a)	6.45	0 (b)	0	0 (b)	0
**Halophilic Bacteria**	**R2A**	0 (a)	0	1.02×10^2 (b)	1.48×10^1	0 (a)	0
	**Marine Agar**	0 (a)	0	7.80×10^1 (b)	4.24	1.60×10^1 (a)	7.85
**Halophilic Yeasts**	**sCMA**	0	0	0	0	0	0
	**sSDA**	0	0	0	0	0	0
	**R2A**	7.20×10^1 (a)	6.52	0 (b)	0	0 (b)	0
	**Marine Agar**	9.60×10^1 (a)	8.13	2.46×10^1 (b)	9.13	0 (c)	0
**Halophilic Moulds**	**sCMA**	0	0	0	0	0	0
	**sSDA**	0	0	0	0	0	0
	**R2A**	0	0	0	0	0	0
	**Marine Agar**	0	0	0	0	0	0

TBC: total bacterial count at 30 °C; sCMA: saline Corn Meal Agar; sSDA: saline Sabouraud Dextrose Agar

Table S7. Microbiological analyses of *Phyllorhiza punctata* JF sample washed with drinking water (JF-DW) and treated with brines containing calcium salts (Ca-Citrate: calcium citrate; Ca-Lactate: calcium lactate). The different letters in line indicate significant differences between samples ($p < 0.05$)

Phyllorhiza punctata							
		**JF-DW**		**Ca-Citrate**		**Ca-Lactate**	
**Microorganisms**	**Medium**	**Mean (CFU/g)**	**SD**	**Mean (CFU/g)**	**SD**	**Mean (CFU/g)**	**SD**
**TBC**	**PCA**	1.08×10^2 (a)	8.74	5.10×10^1 (b)	2.34	7.12×10^1 (b)	1.10×10^1
***Bacillus* spp.**	**BCSA**	1.20×10^1 (a)	6.98	1.20×10^1 (a)	3.61	0 (b)	0
**H_2S-producing bacteria**	**IRON AGAR**	1.80×10^1 (a)	4.26	0 (b)	0	0 (b)	0
**Enterobacteriaceae**	**VRBGA**	0	0	0	0	0	0
***Coli-Aerogenes* Bacteria**	**VRBA**	0	0	0	0	0	0
**Coagulase positive staphylococci**	**Baird Parker Agar**	0	0	0	0	0	0
***Pseudomonas* spp.**	**Pseudomonas Agar**	0	0	0	0	0	0
**Yeasts**	**DRBC**	0	0	0	0	0	0
**Moulds**		0	0	0	0	0	0
**Halophilic Bacteria**	**R2A**	1.68×10^2 (a)	5.09×10^1	1.50×10^1 (b)	2.12	2.46×10^2 (a)	$3.81 \times 10_1$
	**Marine Agar**	6.60×10^1 (a)	4.08	2.70×10^1 (b)	2.44	1.02×10^2 (c)	1.27×10^1
**Halophilic Yeasts**	**sCMA**	0 (a)	0	9.05 (b)	2.32	0 (a)	0
	**sSDA**	0	0	0	0	0	0
	**R2A**	0	0	0	0	0	0
	**Marine Agar**	0	0	0	0	0	0
**Halophilic Moulds**	**sCMA**	0	0	0	0	0	0
	**sSDA**	0 (a)	0	1.20×10^1 (b)	4.06	0 (a)	0
	**R2A**	0	0	0	0	0	0
	**Marine Agar**	0 (a)	0	1.50×10^1 (b)	2.16	0 (a)	0

TBC: total bacterial count at 30°C; sCMA: saline Corn Meal Agar; sSDA: saline Sabouraud Dextrose Agar

Table S8. Texture, salinity and pH values of *Cotylorhiza tuberculata. Rhopilema nomadica* and *Phyllorhiza punctata* JF samples washed with drinking water (JF-DW) and treated with brines containing calcium salts at 5 days treatment (Ca-Citrate: calcium citrate; Ca-Lactate: calcium lactate). The different letters in line indicate significant differences between samples ($p < 0.05$)

	Texture (N)			Salinity (% NaCl)			pH		
	JF-DW	Ca-Citrate	Ca- Lactate	JF-DW	Ca-Citrate	Ca- Lactate	JF-DW	Ca-Citrate	Ca- Lactate
C. tuberculata	-16.6± 4 (a)	-34.9± 5 (b)	-29.6 ± 8 (b)	2	1	1.2	7.9	7.1	7.2
R. nomadica	-63.3 ± 9 (a)	-27.2 ± 3 (b)	-30.7 ± 9 (b)	3.5	1	1	7.9	7	6
P. punctata	-47.2 ± 9 (a)	-24.3 ± 3 (b)	-22.7 ± 8 (b)	1.2	0.5	1	7.9	6	6.8

Table S9. Comparison of the fatty acid composition of and *Rhopilema nomadica. Phylloriza punctata* and *Cothyloriza tuberculata* JF samples washed with drinking water (JF-DW) and treated with brines containing calcium salts at 5 days treatment (Ca-Citrate: calcium citrate; Ca-Lactate: calcium lactate). Fatty acid composition data are expressed as percentage of the total fatty acids ± SD.

	Fatty acids composition (%)								
	R. nomadica JF-DW			*P. punctata* JF-DW			*C. tuberculata* JF-DW		
	JF-DW	Ca-Citrate	Ca-Lactate	JF-DW	Ca-Citrate	Ca-Lactate	JF-DW	Ca-Citrate	Ca-Lactate
**Saturated FA (SFA)**									
**Myristic acid $C_{14:0}$**	6.6 ± 0.7	—	9.2 ± 0.2	10.5 ± 1.1	8.4 ± 0.8	7.7 ± 0.8	—	—	—
**Pentadecanoic acid $C_{15:0}$**	—	—	—	2 ± 0.4	—	—	—	—	—
**Palmitic acid $C_{16:0}$**	32.4 ± 4.0	44.2 ± 4.5	38.8 ± 0.4	28.3 ± 3.0	20.9 ± 2.0	20.1 ± 2.0	44.7 ± 4.5	43.2 ± 4.0	41.2 ± 3.3
**Margaric acid $C_{17:0}$**	2.6 ± 0.2	1.6 ± 0.2	—	1.4 ± 0.2	2.5 ± 0.2	2.3 ± 0.2	—	—	—
**Stearic acid $C_{18:0}$**	26.0 ± 3.9	37.6 ± 4.0	29.8 ± 2.8	15.2 ± 3.5	14.6 ± 1.5	14.2 ± 2.7	34.4 ± 4.0	38.8 ± 3.8	35.2 ± 3.4
**Nonadecanoic acid $C_{19:0}$**	—	—	—	—	—	—	—	13.2 ± 1.3	—
**Arachidic acid $C_{20:0}$**	2.2 ± 0.2	5.1 ± 0.5	1.4 ± 0.2	—	1.5 ± 0.2	1.2 ± 0.3	—	—	1.9 ± 0.2
**Total SFA**	**69.8 ± 7.0**	**88.5 ± 8.9**	**79.2 ± 8.0**	**57.3 ± 5.7**	**48.0 ± 4.8**	**45.5 ± 4.5**	**79.1 ± 8.1**	**95.2 ± 8.2**	**78.3 ± 7.8**
**Monounsaturated FA (MUFA)**									
**Palmitoleic acid $C_{16:1}$ (ω7)**	—	—	—	5.0 ± 0.5	4.4 ± 0.5	4.0 ± 0.4	—	—	—
**Oleic acid $C_{18:1}$ (ω9)**	3.1 ± 0.2	3.3 ± 0.4	3.7± 0.4	4.3 ± 0.3	4.8 ± 0.5	—	—	—	—
**Isooleic acid $C_{18:1\ trans\text{-}10}$**	2.3 ± 0.4	—	1.2 ± 0.1	—	—	6.7 ± 0.3	—	—	8.6 ± 0.9
**Vaccenic acid $C_{18:1\ cis\text{-}11}$ (ω7)**	—	—	—	1.5 ± 0.2	2.2 ± 0.2	—	—	—	—

**Total MUFA**	**5.4 ± 0.5**	**3.3 ± 0.4**	**4.9 ± 0.5**	**10.8 ± 0.3**	**11.4 ± 0.7**	**10.7 ± 0.3**	**0**	**0**	**8.6 ± 0.9**
**Polyunsaturated FA (PUFA)**									
**Linoleic acid $C_{18:2}$ (ω6)**	2.3 ± 0.3	—	6.9 ± 0.7	4.8 ± 0.5	2.9 ± 0.3	2.8 ± 0.3	—	—	—
**Isolinoleic acid $C_{18:2\ trans\ 8,11}$**	—	—	—	—	—	—	—	—	11.1 ± 1.1
**Linolenic acid $C_{18:3}$ (ω3)**	2.7 ± 0.4	—	—	3.3 ± 0.3	4.2 ± 0.4	4.9 ± 0.5	—	—	—
**Stearidonic acid $C_{18:4}$ (ω3)**	2.0 ± 0.2	—	—	3.8 ± 0.4	4.2 ± 0.4	4.0 ± 0.4	7.5 ± 0.8	—	—
**Eicosadienoic acid $C_{20:2}$ (ω6)**	—	—	—	—	—	0.6 ± 0.2	—	—	—
**Arachidonic acid $C_{20:4}$ (ω6)**	7.4 ± 0.7	4.6 ± 0.5	5.0 ± 0.4	3.8 ± 0.4	7.9 ± 0.5	5.5 ± 0.6	4.4 ± 0.3	2.0 ± 0.1	2.0 ± 0.2
**Eicosapentaenoic acid $C_{20:5}$ (ω3)**	5.6 ± 0.6	1.5 ± 0.2	1.0 ± 0.5	8.5 ± 0.6	9.5 ± 0.8	13.2 ± 1.0	4.1 ± 0.3	1.9 ± 0.2	—
**Docosapentaenoic acid $C_{22:5}$ (ω3)**	0.9 ± 0.1	—	—	0.9 ± 0.1	1.6 ± 0.1	1.8 ± 0.2	—	—	—
**Docosahexaenoic acid $C_{22:6}$ (ω3)**	4.2 ± 0.4	2.1± 0.2	3.0 ± 0.3	6.8 ± 0.6	10.2 ± 0.2	10.9 ± 1.1	5.0 ± 0.5	0.9 ± 0.1	—
**Total PUFA**	**25.0 ± 2.6**	**8.2 ± 0.8**	**15.9 ± 1.3**	**34.6 ± 3.2**	**40.6 ± 4.1**	**43.8 ± 4.4**	**20.9 ± 0.8**	**4.8 ± 0.5**	**13.1 ± 1.3**
**Σω6**	9.6	4.6	11.9	8.6	10.8	9.0	4.4	2	2
**Σω3**	15.4	3.7	4.0	23.2	29.8	34.8	16.5	2.7	-
**ω6/ω3**	0.6	1.2	3.0	0.4	0.4	0.3	0.3	0.7	-
**Total Lipids (%DW)**	26.5	72.0	68.6	83.8	56.5	72.4	29.0	12.5	4.0
